# Peripheral neuropathy after viral eradication with direct‐acting antivirals in chronic HCV hepatitis: A prospective study

**DOI:** 10.1111/liv.15002

**Published:** 2021-07-20

**Authors:** Maria M. Zanone, Claudia Marinucci, Alessia Ciancio, Dario Cocito, Federica Zardo, Emanuela Spagone, Bruno Ferrero, Cristina Cerruti, Lorena Charrier, Franco Cavallo, Giorgio M. Saracco, Massimo Porta

**Affiliations:** ^1^ Internal Medicine 1 Department of Medical Sciences University of Turin Torino Italy; ^2^ Division of Gastroenterology and Hepathology Department of Medical Sciences University of Turin Torino Italy; ^3^ Department of Neurosciences University of Turin Torino Italy; ^4^ Department of Public Health and Paediatrics University of Turin Torino Italy

**Keywords:** cryoglobulinemia, direct‐acting antiviral agents, hepatitis C virus, peripheral neuropathy, quality of life

## Abstract

**Background:**

HCV‐related extra‐hepatic complications include peripheral neuropathies, with important prevalence and impact. A recent metanalysis of previous intervention trials concluded for insufficient data to support evidence‐based treatments for this complication. In this longitudinal study, we assessed for the first time prevalence and outcome of neuropathy in a cohort of patients with chronic HCV, before and after direct‐acting antiviral agent (DAA) treatment.

**Method:**

Ninety‐four patients (mean age 58.5 ± 9.9, infection duration 22.2 ± 6.3 years) without systemic and metabolic diseases, underwent neurological examination and electroneurography studies before (T0) and 10.4 ± 1.7 months after the end of DAA therapy (T1), and cryoglobulins (CG) assessment. Muscle strength was evaluated by Medical Research Council (MRC) score; neuropathic pain, sensory function, disability, quality of life were assessed by validated questionnaires (DN4, NPSI, SSS, INCAT and Euro‐QoL).

**Results:**

At T0, sensory‐motor neuropathy was detected in 22 patients (23%), reflexes were depressed in 32 (34%) with no association with infection duration, viral load, age, CG. Neuropathic pain (DN4 ≥4) was present in 37 patients (39%). At T1, out of the 22 patients with altered electroneurography, 3 had died or developed HCC, 4 showed normal electroneurography, and nerve amplitude parameters tended to improve in the whole group. Only 11 patients (12%) had depressed reflexes and 10 (11%) DN4 ≥4 (*P* < .05 compared to T0). Scores for MRC, questionnaires and Euro‐QoL improved significantly (*P* < .05).

**Conclusion:**

Our study confirms the high prevalence of clinical and subclinical peripheral sensory‐motor neuropathy in patients with HCV infection and indicates improvement after eradication by DAA. These results support the need for larger intervention studies.

**Abbreviations**
CG: Cryoglobulins
CMAP: Compound muscle action potential
DAAs: Direct‐acting antiviral agents
DL: Distal latency
DN4: Douleur Neuropathique 4
HCC: Hepatocellular carcinoma
HCV: Hepatitis C virus
INCAT: Inflammatory Neuropathy Cause and Treatment
MCV: Motor conduction velocity
MRC: Medical Research Council
NPSI: Neuropathic Pain Symptom Inventory
Qol: Quality of life
SCV: Sensory conduction velocity
SNAP: Sensory nerve action potential
SSS: Sensory Sum Score

**Key points**

- Chronic HCV infection carries a series of extra‐hepatic complications occurring early and even more frequently than those related to hepatic involvement.
- These extra‐hepatic complications include neurological damage at various levels, with high prevalence and clinical impact.
- This longitudinal study shows that successful HCV eradication with DAAs can improve neurological parameters, as well as quality of life.

## 1 INTRODUCTION

Hepatitis C virus (HCV) infection is a major cause of morbidity and mortality, with a strong socio‐economic impact. The WHO estimates that 1.1% of the global population has HCV, with wide geographic distribution and potential underestimation as silent infections progress asymptomatically for years. An estimated 1.4% of the population in the US and an estimated 1.25%‐1.75% in Italy has HCV, reaching 20% in people above 70 years of age^1^ in some areas. HCV infection is associated with several extrahepatic manifestations that increase morbidity and mortality, decrease quality of life^2^, ^3^ and may contraindicate antiviral therapy, especially with interferon α (IFN‐α). On the other hand, successful eradication of HCV with IFN‐ α and ribavirin was accompanied by improvement of some of these manifestations, with possible resolution in case of sustained virological response, as in the case of mixed cryoglobulinemia.^4^, ^5^

Extrahepatic comorbidities of chronic HCV infection include neurological complications, involving both the central (fatigue, cognitive impairment) and the peripheral nervous system.^6^, ^7^, ^8^ Peripheral neuropathies, cryoglobulinemic or non‐cryoglobulinemic are the most common neurological complications, with a prevalence of up to 86% of infected patients with, and 43.5% of those without, cryoglobulinemia. Prevalence, however, varies depending on the study population, the definition and method of assessment of neuropathy, including electrophysiological studies and standard questionnaires.^4^, ^8^, ^9^ For instance, standard electrophysiological studies detected peripheral neuropathy in 15.3% of patients, subclinical in approximately one‐third of them.^9^ Prevalence rises to 90% when considering only subjective symptoms, such as paraesthesias, among patient with mixed cryoglobulinemia.^10^

There is, at present, insufficient evidence to support treatment of HCV‐related neuropathy and therapeutic approaches may differ, depending on the presence of cryoglobulinemia and the different potential pathogenetic mechanisms underlying nerve damage.^11^, ^12^ A recent metanalysis of all intervention trials, including treatments with IFN‐α, ribavirin, corticosteroids, cyclophosphamide, plasma exchange, and rituximab, alone or in combination, failed to show improvement of HCV‐associated peripheral neuropathy up to 36 months post‐treatment, while demonstrating potential adverse events.^13^ Furthermore, there are no reliable studies evaluating treatment of non‐cryoglobulinemic neuropathy associated with HCV infection.

A new era started in 2013 with the introduction of direct‐acting antiviral agents (DAAs), achieving HCV eradication rates of approximately 95% with reduced side effects compared to previous classical treatments.^14^ Recent studies report favourable outcomes for extrahepatic HCV‐related complications,^5^ including insulin resistance, glycaemic control^15^ or endothelial function and cardiovascular morbidity.^16^, ^17^ However, the impact of HCV eradication achieved by DAAs on HCV‐related neuropathies has not been explored systematically, and some data are available only in the context of cryoglobulinemic vasculitis.^5^ Two case reports of greatly improved neuropathy after diagnosis and eradication of hitherto ignored HCV infection are suggestive of favourable outcomes of DAA therapy on this complication.^18^, ^19^

In the present study, we aimed to assess the prevalence of peripheral neuropathy associated with HCV‐infection with and without cryoglobulinemia, and to evaluate prospectively the effects of HCV eradication by DAAs, using a global assessment by standard nerve conduction studies, neurological examination, together with validated questionnaires exploring neurological motor and sensory symptoms, disability and quality of life.

## 2 METHODS

### 2.1 Patients

Out‐patients with a history of chronic HCV‐infection, younger than 75, and eligible to start treatment with DAAs according to the EASL guidelines^20^ attending the Hepatic Clinic at Turin University, were consecutively considered for participation in the study. Exclusion criteria included non compensated or advanced cirrhosis, current, and history of, alcohol or drug abuse, smoking, clinical history of diabetes or altered fasting glucose, hypothyroidism, connective tissue diseases or other forms of chronic arthritis/artropathy, vertebral discopathy, entrapment mononeuropathies, chronic kidney disease (stage IV‐V), past or current malignancy. Further clinical information on BMI, previous anti‐viral therapies (interferon, ribavirin), medication intake, viral genotype and load was obtained from medical records. The severity of liver disease was graduated according to the degree of liver fibrosis defined on Metavir stage, as estimated by FibroScan^21^ and Child‐Pugh score was calculated.^22^ Blood samples were obtained for serum chemistry profile and cryoglobulin (CG) determination. Briefly, blood samples were kept at 37℃ for 30 minutes before separation. Serum was prepared by centrifuging at 37℃ for 10 minutes at 1245 g. The serum obtained was transferred to a 15‐mL glass graduated conical tube, and incubated at 4℃ for 7 days. If a precipitate was detected, the tube was centrifuged at 1245 g for 30 minutes at 4℃. The cryoprecipitate was visually measured according to the graduated level of the glass tube and expressed as a percentage of precipitate/serum volume.

Serum samples were frozen at −20℃ for subsequent studies. Determination of HCV genotype with viral load were assessed and patients were treated according to guidelines for HCV infection,^20^ and re‐evaluated 10.4 ± 1.7 months (T1) after the ending of the 8 or 12 weeks course of DAA therapy, depending on DAA.

Informed consent was obtained and the investigations carried out in conformity with the Declaration of Helsinki. The study was approved by the local Ethics Committee.

### 2.2 Assessment of neuropathy and quality of life

At T0, all participants received a full clinical and neurological examination, including deep tendon knee and ankle reflexes assessment and recording of vibratory perception threshold at the tip of the big toe, using a 128 Hz tuning fork. Electrophysiological tests were performed by standard equipment (Dantec^™^ Keypoint^®^ G4, Natus Neurology Incorporated). Skin temperature was maintained at 36℃ using infrared heating when needed, and nerve conduction studies were performed according to standard techniques.^23^ Compound muscle action potential (CMAP) and sensory nerve action potential (SNAP) amplitudes were recorded by surface electrodes. Motor conduction velocity (MCV), distal latency (DL) and CMAP amplitude (baseline to negative peak) were measured in the median, ulnar, and peroneal nerves of both sides. Sensory nerve conduction velocity (SCV) and SNAP amplitude were measured in the median, ulnar, and sural nerves of both sides. Tests were performed according to standard techniques indicated by Kimura,^24^ and the normality cut‐off values adopted were derived from the mean ± SD of a group of 73 healthy control subjects studied in our laboratory (mean age 55.5 ± 12.7 years). Results were classified as normal, or as altered if any parameter of the nerve assessed was not within the control mean −2SD (for CAMP, MCV, SAP, SCV) or control mean +2SD (for DL).

Muscle strength was tested by the Medical Research Council (MRC) scale, which evaluates the strength of upper and lower limb movements, ranging from 0 (no contraction) to 5 (normal strength) and then adding the obtained values, according to MRC sum scores criteria.^25^ Scores ranged from 0 to 80.

Structured validated questionnaires were used to identify symptoms related to motor and sensory function, as typically used for primary endpoints in inflammatory polyneuropathy clinical trials. Specifically, neuropathic pain (scale and number of reporting patients), impairment (sensory and motor scale), disability (scale), global impression of change according to improved or resolved symptoms, impacting on quality of life were evaluated.

The Douleur Neuropathique 4 (DN4)^26^ tool evaluates neuropathic pain through 10 items: 7 evaluating pain quality as defined by the patient and 3 based upon clinical assessment of hypoaesthesia to touch and pinprick and allodynia. Total score derives from the sum of all 10 items and the cut‐off value for neuropathic pain diagnosis is 4/10.^26^DN4 has 82%‐83% sensitivity, and 81%‐90% specificity.^26^, ^27^

The Neuropathic Pain Symptom Inventory (NPSI)^28^ is a self‐administered questionnaire assessing spontaneous ongoing or paroxysmal pain, evoked pain and dysesthesia/paraesthesia. It is structured in 12 items, 10 on different symptoms, and 2 on duration, each quantified on a numerical scale ranging 0‐10; total score was recorded.

The Sensory Sum Score (SSS)^29^ comprises pin prick and vibration sense plus a two point discrimination value in the arms and legs, and ranges from 0 (“normal sensation”) to 20 (“most severe sensory deficit”).

The Inflammatory Neuropathy Cause and Treatment (INCAT)^30^ tool measures disability of upper and lower limbs, testing the ability to use either arm for purposeful movements and to walk without support or wheelchair, respectively. Scores ranged from 0 (no disability) to 12 (severe disability).

Quality of life was evaluated using the Euro‐QoL,^31^ based upon self‐evaluation of health‐related quality of life assessing mobility, self‐care, usual activities, pain/discomfort, and anxiety/depression, each with 3 levels of increasing severity. Index is then calculated subtracting from 1, the best health state, standard coefficients that increase with gravity.

Investigators studying clinical features were unaware of electrophysiology and lab testing results, as were the other investigators of the clinical data.

At T1, all patients were re‐examined and questionnaires administered, and only those with abnormal electroneurography at baseline underwent a new electrophysiological study.

### 2.3 Statistical analysis

Data are shown as absolute and relative (%) frequencies for categorical data and mean ± SD for continuous variables.

Mc Nemar test for categorical variables and paired *t* test or Wilcoxon signed‐rank test, as appropriate, were carried out to detect significant differences between baseline and follow‐up data for neurological characteristics of all patients and for electrophysiological data for patients who showed abnormal results at baseline.

Chi‐square test for categorical variables and *t* test for continuous variables, or Wilcoxon rank‐sum test in case of nonparametric distribution, were performed to assess whether significant differences for demographic and clinical data could be found between patients with and without abnormal electrophysiological results at baseline.

Chi‐square test for categorical variables and *t* test for continuous variables, or Wilcoxon rank‐sum test, as appropriate, were carried out to assess whether significant differences could be evidenced between CG+and CG‐ groups for socio‐demographic, clinical and neurological data at baseline.

Differences between baseline and T1 for neurological data were then tested in both groups (GC+and GC‐) with paired *t* test or Wilcoxon signed‐rank test for continuous variables and Mc Nemar test for categorical variables. Differences between baseline and T1 (∆) for neurological data were finally compared between CG+and GC‐ by means of chi‐square test for categorical variables and *t* test or Wilcoxon rank‐sum test for continuous variables, as appropriate.

For all tests, a *P*‐value of less than 5% was considered significant.

All analyses were performed with Stata 14.

## 3 RESULTS

### 3.1 HCV‐related neuropathy

Ninety‐four patients were recruited at baseline (T0) and their clinical characteristics are summarised in Table 1. Forty‐seven patients (50%) were treated with Sofosbuvir/Velpatasvir (Epclusa^®^) for 12 weeks, 29 (31%) with Glecaprevir/Pibrentasvir (Maviret^®^) for 8 weeks, and the remaining ones (19%) with Elbasvir/Grazoprevir (Zepatier^®^) for 12 weeks, according to clinical and virological variable.^32^

**TABLE 1:** Clinical characteristics of HCV‐positive patients at recruitment (T0)

Clinical characteristics	T0 (n = 94)
Sex (M/F)	47/47
Age (years)	58.5 ± 9.9
Body Mass Index	24.4 ± 3.8
Duration of disease (years)	22.2 ± 6.3
DAA Treatment	
Sofosbuvir/Velpatasvir	47 (50.0)
Glecaprevir/Pribrentasivir	29 (30.9)
Elbasvir/Grazoprevir	18 (19.1)
Comorbidities	
0	21 (22.4)
1	27 (28.7)
2	46 (48.9)
Concomitant therapies	
0	32 (34.0)
1	61 (64.9)
2	1 (1.1)
Previous IFN‐α therapy	27 (29.0)
Child‐Pugh	
A	90 (95.7)
B	4 (4.3)
Fibrosis	81 (86.2)
Creatinine (mg/dL)	0.76 ± 0.18
CG+	28 (32.2)
HCV Genotype	
1	64 (68.1)
2	12 (12.8)
3	11 (11.7)
4	7 (7.4)

Note

Data are expressed as n (%), or mean ± SD.

Cryoglobulins, CG, were assessed in 87 patients.

Comorbidities include hypertension, dyslipidemia, osteoporosis, chronic kidney disease (stage I‐II).

**FIGURE 1:**
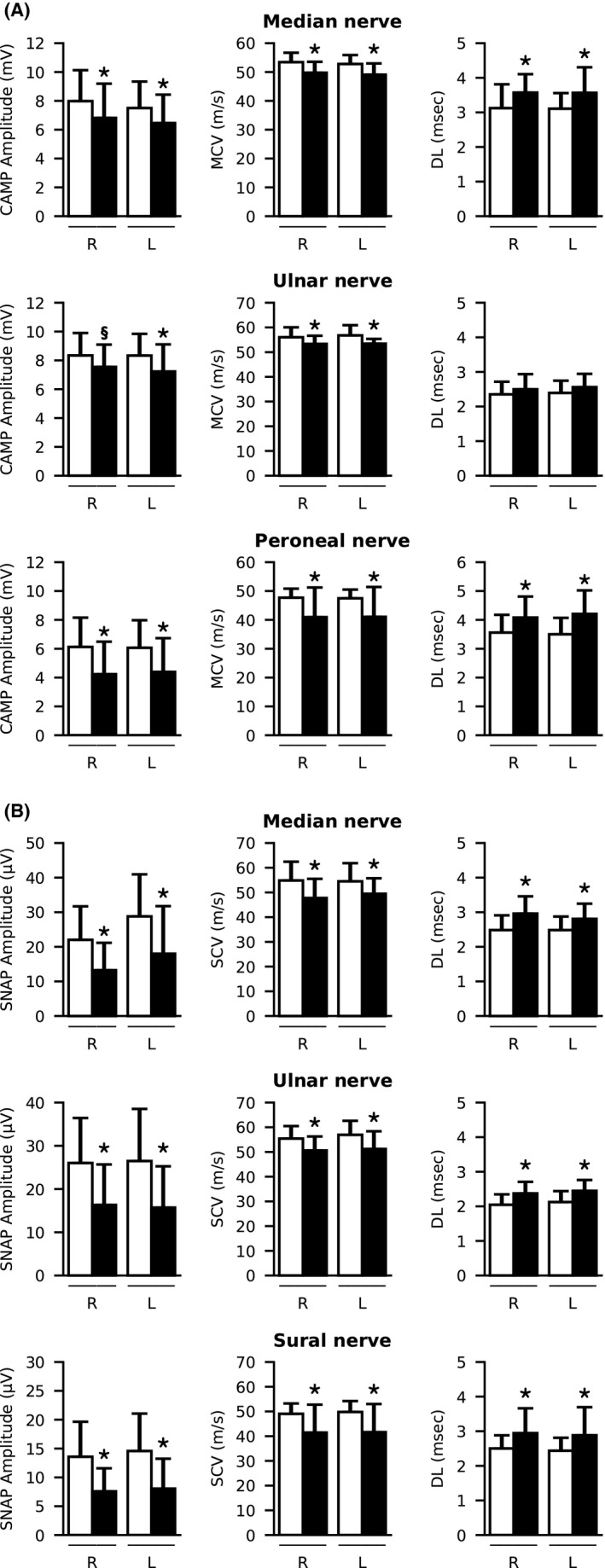
Electrophysiological data at T0. Mean±SD values of CAMP amplitude, MCV and DL for motor nerves (A), and of SNAP amplitude, SCV and DL for sensory nerves (B) in 72 patients with normal parameters (white) and 19 patients with neuropathy (black) detected at T0. *P < .05 vs patients with normal parameters. ^§^ *P =* .051 vs patients with normal parameters

At T0, nerve conduction studies disclosed moderate to severe, length‐dependent, sensori‐motor or predominantly sensory axonal neuropathy, prevalent symmetrical on the lower limbs, in 22 patients (23%), with no significant differences in age, duration of infection, creatinine, viral load, viral genotype, grade of liver fibrosis, or presence of concomitant pathologies compared to the patients with normal electrophysiological results (Figure 1). Thirty‐two (34%) showed impaired deep tendon reflexes (Table 2), with higher prevalence in patients with altered electroneurography, compared to those with normal results (18/22, 82% vs 14/72, 19%, *P* < .01). Questionnaire scores are shown in Table 2. Thirty‐seven patients (39%) complained of neuropathic pain at DN4, 15 (16%) had severe sensory symptoms and 24 (26%) severely impaired muscle strength.

**TABLE 2:** Neurological characteristics of HCV‐positive patients at recruitment (T0) and comparison 10 months after DAA treatment (T1)

Neurological characteristics	T0 (n = 94)	T1 (n = 91)	*P‐*value
Absent/depressed reflexes	32 (34.0)	11 (12.1)	**<.000**
DN4			
Mean (SD)	3.11 (2.19)	1.23 (1.74)	**<.000**
Median (IQR)	3 (1‐4)	1 (0‐2)	
DN4 ≥4, n (%)	37 (39.4)	10 (11.0)	**<.000**
NPSI			
Mean (SD)	12.0 (13.7)	5.0 (9.0)	**<.000**
Median (IQR)	7.5 (2‐18)	0 (0‐6)	
SSS			
Mean (SD)	4.4 (4.4)	2.1 (3.1)	**<.000**
Median (IQR)	3.5 (0‐8)	0 (0‐4)	
SSS ≥8, n (%)	15 (16.0)	4 (4.4)	.**0075**
INCAT			
Mean (SD)	1.33 (1.5)	0.47 (0.72)	**<.000**
Median (IQR)	1 (0‐2)	0 (0‐1)	
Euro‐Qol			
Mean (SD)	0.68(0.37)	0.89 (0.18)	**<.000**
Median (IQR)	0.80 (0.49‐1)	1 (0.85‐1)	
MRC			
Mean (SD)	73.1 (9.7)	77.0 (5.0)	**<.000**
Median (IQR)	76 (70‐80)	79 (77‐80)	
MRC ≤70, n (%)	24 (25.5)	7 (7.7)	.**0001**

Note

MRC ≤70, lower quartile; SSS ≥8, upper quartile.

Significant *p*‐values are in bold.

Abbreviations: DN4, Douleur Neuropathique 4; INCAT, Inflammatory Neuropathy Cause and Treatment; MRC, Medical Research Council; NPSI, Neuropathic Pain Symptom Inventory; Qol, Quality of life; SSS, Sensory Sum Score.

There was a trend towards higher prevalence of altered nerve conduction studies, worse amplitude parameters (significantly different for median nerve CMAP amplitude, 6.7 ± 2.4 vs 8.4 ± 1.9 mV, *P* = .001, and peroneal nerve CMAP amplitude, 4.8 ± 2.2 vs 6.1 ± 2.1 mV, *P* = .012) and worse neurological symptoms in CG+compared to CG‐ patients, with only SSS reaching a statistically significant difference (*P* = .031) (Table 3).

**TABLE 3:** Clinical and neurological characteristics of HCV‐positive patients at recruitment (T0) according to presence or absence of cryoglobulins (CG)

	CG+ n = 28	CG‐ n = 59	*P‐*value
Altered electroneurography	9 (32.1)	13 (22.0)	.429
Absent/altered reflexes	11 (39.3)	20 (33.9)	.624
Sex (M/F)	10/18	34/25	.056
Age (years)	62.4 ± 10.0	57.3 ± 9.4	.**026**
Body Mass Index	24.2 ± 3.4	24.7 ± 4.0	.949
Duration of disease (years)	22.4 ± 6.4	22.2 ± 6.2	.878
Comorbidities (0/1/2)	3/8/17	15/16/28	.266
Concomitant therapies	17 (60.7)	42 (71.2)	.329
Creatinine (mg/dl)	0.74 ± 0.19	0.78 ± 0.17	.216
Previous IFN‐α therapy	8 (29.6)	17 (28.8)	.938
Child‐Pugh			
A	26	57	.591
B	2	2	
Fibrosis	23 (82.1)	51 (86.4)	.749
HCV Genotype			
(1/2/3/4)	17/7/3/1	43/4/6/6	.106
DN4			
Mean (SD)	3.5 (2.5)	2.8 (2.0)	.190
Median (IQR)	3 (2‐5)	3 (1‐4)	.254
DN4 ≥4, n (%)	13 (46.4)	19 (32.2)	.199
NPSI			
Mean (SD)	12.7 (14.7)	11.6 (14.0)	.732
Median (IQR)	7.5 (1.5‐21)	7 (0‐17)	.724
SSS			
Mean (SD)	5.7 (4.7)	3.6 (4.0)	.**0316**
Median (IQR)	5.5 (1.5‐8.5)	2 (0‐7)	.**0310**
SSS ≥8, n (%)	6 (21.4)	7 (11.9)	.334
INCAT			
Mean (SD)	1.78 (2.0)	1.12 (1.2)	.057
Median (IQR)	1 (0.5‐2.5)	1 (0‐2)	.153
EuroQol			
Mean (SD)	0.57 (0.52)	0.74 (0.27)	.**047**
Median (IQR)	0.7 (0.48‐0.85)	0.8 (0.64‐1)	.111
MRC			
Mean (SD)	70.6 (13.3)	74.3 (7.3)	.104
Median (IQR)	75 (67.5‐78)	77 (72‐80)	.112
MRC ≤70, n (%)	8 (28.6)	14 (23.7)	.792

Note

Data are expressed as n (%), or mean ± SD.

Significant *p*‐values are in bold.

Cryoglobulins, CG, were assessed in 87 patients.

Abbreviations: CG, Cryoglobulins; DN4, Douleur Neuropathique 4; INCAT, Inflammatory Neuropathy Cause and Treatment; MRC, Medical Research Council; NPSI, Neuropathic Pain Symptom Inventory; Qol, Quality of life; SSS, Sensory Sum Score.

Considering the prognostic index, patients with Child Pugh B showed significantly worse MRC (53 ± 25.3 vs 73.9 ± 7.5, *P* = .008), DN4 (5.4 ± 1.8 vs 3 ± 2.1, *P* = .0408), NPSI (26.5 ± 19.3 vs 11.4 ± 13.2, *P* = .033), SSS (11 ± 5.5 vs 4.1 ± 4.1, *P* = .014), and INCAT (4.3 ± 3.3 vs 1.2 ± 1.3, *P* = .009) scores compared to grade A, while lower Euro‐QoL approached significance (0.003 ± 1.1 vs 0.71 ± 0.3, *P* = .06).

### 3.2 Re‐assessment after DAA treatment

At the end of treatment, all patients but one showed undetectable levels of HCV‐RNA. Ninety‐one patients completed the 10 months follow‐up after the end of treatment. At T1, 3 of the patients with abnormal electroneurography dropped out (1 sudden death, 2 developed HCC), while 4 of the 19 patients with sensory‐motor neuropathy completing the study (4/19, 21%) showed a normal electroneurography (Figure S1), and 2 had a marked improvement of nerve amplitude parameters (1 patient with absent peroneal nerve CAMP recovered motor response, although still abnormal, and 1 patient approached normal parameters for sural SNAP).

In general, there was a trend towards an increase of motor and sensory amplitude parameters in the whole subgroup, reaching or approaching statistical significance for median, peroneal nerve CMAP and sural nerve SNAP amplitudes, mainly for CG‐ patients (Table 2 and 4). Deep tendon reflexes remained impaired in only 11 patients (11/91, 12%) (*P* < .000 compared to T0), and 10 patients (10/91, 11%) still complained neuropathic pain at DN4 (*P* < .000 compared to T0). There was a statistically significant improvement for all neurological scores, indicating significant improvements for pain, limb disability, muscle strength, and global impression of quality of life (*P* < .000 compared to T0) (Table 2).

**TABLE 4:** Electrophysiological data for motor and sensory nerve conduction studies in the 19 patients with neuropathy detected at T0 and re‐evaluated at T1. Data are expressed as mean (SD); *P*‐value is the result of a paired *t* test between T0 and T1

	T0 (n = 19)	T1 (n = 19)	*P‐*value
Median nerve			
CMAP Amplitude (mV)			
R	6.8 (2.4)	7.45 (1.8)	.0912
L	6.5 (2.0)	7.3 (1.5)	.**0123**
MCV (m/sec)			
R	49.8 (3.8)	49.3 (4.3)	.529
L	49.1 (3.9)	49.8 (4.1)	.361
DL (msec)			
R	3.6 (0.5)	3.6 (0.7)	.795
L	3.6 (0.7)	3.6 (0.9)	.776
Ulnar nerve			
CMAP Amplitude (mV)			
R	7.5 (1.5)	8.0 (1.5)	.159
L	7.2 (1.9)	7.4 (1.3)	.582
MCV (m/sec)			
R	53.3 (3.3)	52.6 (4.1)	.308
L	53.4 (1.9)	52.7 (2.9)	.335
DL (msec)			
R	2.5 (0.4)	2.7 (0.5)	.1464
L	2.6 (0.4)	2.7 (0.4)	.**0213**
Peroneal nerve			
CMAP Amplitude (mV)^a^			
R	4.2 (2.2)	4.6 (2.5)	.063
L	4.4 (2.3)	4.7 (2.0)	.093
MCV (m/sec)			
R	41.0 (10.3)	40.6 (10.1)	.366
L	41.0 (10.4)	43.2 (3.4)	.267
DL (msec)			
R	4.1 (0.7)	4.3 (1.0)	.446
L	4.2 (0.8)	4.2 (0.9)	.923
Median nerve			
SNAP Amplitude (μV)			
R	13.3 (7.9)	16.0 (11.2)	.190
L	18.0 (13.7)	17.8 (12.1)	.189
SCV (m/sec)			
R	47.8 (7.7)	49.6 (8.1)	.163
L	49.5 (6.2)	48.8 (7.1)	.390
DL (msec)			
R	3.0 (0.5)	2.9 (0.5)	.541
L	2.8 (0.4)	3.0 (0.5)	.083
Ulnar nerve			
SNAP Amplitude (μV)			
R	16.3 (9.3)	17.9 (7.9)	.177
L	15.7 (11.9)	16.3 (9.0)	.496
SCV (m/sec)			
R	50.6 (5.6)	49.3 (7.1)	.303
L	51.2 (7.2)	49.2 (8.3)	.**045**
DL (msec)			
R	2.4 (0.3)	2.5 (0.4)	.209
L	2.4 (0.3)	2.5 (0.4)	.279
Sural nerve			
SNAP Amplitude (μV)^b^			
R	7.6 (4.0)	8.9 (4.3)	.**052**
L	8.1 (5.2)	9.4 (4.5)	.070
SCV (m/sec)			
R	41.4 (11.3)	40.7 (11.1)	.459
L	41.6 (11.4)	41.7 (11.5)	.957
DL (msec)			
R	2.9 (0.7)	2.8 (0.7)	.178
L	2.9 (0.8)	2.6 (0.6)	.091

Abbreviations: CMAP, Compound muscle action potential; DL, Distal latency; L, left limb; MCV, Motor conduction velocity; R, right limb; SCV, Sensory conduction velocity; SNAP, Sensory nerve action potential.

Significant *p*‐values are in bold.

^a^ CG‐ patients: T0 4.7 (2.2), T1 5.7 (2.2), ***P* = .034**.

^b^ CG‐ patients: T0 8.5 (3.9), T1 10.6 (4), ***P* = .047**.

When considering the presence of cryoglobulinemia, a significant improvement was detected in both groups (CG+and CG‐) from T0 to T1 for all neurological scores. The improvement in the CG+patient subgroup was also significantly higher than the one in the CG‐ subgroup for neuropathic pain at DN4 (Table 5).

**TABLE 5:** Intra‐ and inter‐group comparison of neurological characteristics of HCV‐positive patients at recruitment (T0) and after DAA treatment (T1), according to presence or absence of cryoglobulins (CG)

Neurological characteristics	CG+ n = 28				CG‐ n = 59*				∆ CG+ vs ∆ CG‐
	T0	T1	∆ (T0‐T1)	*P‐*value	T0	T1	∆ (T0‐T1)	*P‐*value	*P‐*value
Absent/depressed reflexes, n (/%)	11 (39.3)	5 (17.9)	–	.**014**	20 (33.9)	5 (8.9)	–	.**0005**	1.00
DN4									
Mean (SD)	3.5 (2.5)	1.1 (1.7)	2.4 (2.1)	**<.00**	2.83 (2.0)	1.23 (1.8)	1.5 (1.7)	**<.000**	.**04**
Median (IQR)	3 (2‐5)	0 (0‐1.5)	2 (1‐4)		3 (1‐4)	0.5 (0‐2)	1 (0.5‐1)		
DN4 ≥4, n (%)	13 (46.4)	2 (7.1)	–	.**001**	19 (32.2)	7 (12.5)	–	.**0045**	.13
NPSI									
Mean (SD)	12.7 (14.7)	3.5 (5.8)	9.2 (10.3)	**<.00**	11.6 (14.0)	5.7 (10.5)	5.5 (7.9)	**<.000**	.18
Median (IQR)	7.5 (1.5‐21)	0 (0‐6)	6 (1.5‐13.5)		7 (0‐17)	0 (0‐5.5)	4 (0‐10)		
SSS									
Mean (SD)	5.7 (4.7)	3.2 (3.4)	2.5 (2.6)	**<.00**	3.6 (4.0)	1.4 (2.5)	2.1 (3.5)	**<.000**	.10
Median (IQR)	5.5 (1.5‐8.5)	2 (0‐5.5)	2 (0.5‐4)		2 (0‐7)	0 (0‐2)	0.5 (0‐3)		
SSS ≥8, n (%)	6 (21.4)	1 (3.6)	–	.**025**	7 (12.0)	1 (1.8)	–	.06	.36
INCAT									
Mean (SD)	1.8 (2.0)	0.6 (0.9)	1.2 (1.6)	**<.00**	1.12 (1.2)	0.4 (0.6)	0.7 (1.0)	**<.000**	.18
Median (IQR)	1 (0.5‐2.5)	0 (0‐1)	1 (0‐1)		1 (0‐2)	0 (0‐1)	0 (0‐1)		
Euro‐Qol									
Mean (SD)	0.6 (0.5)	0.9 (0.1)	−0.3 (0.5)	**<.00**	0.74 (0.27)	0.89 (0.2)	−0.15 (0.2)	**<.000**	.07
Median (IQR)	0.7 (0.5‐0.8)	0.9 (0.8‐1)	−0.2 (−0.4;0)		0.8 (0.6‐1)	1 (0.8‐1)	−0.04 (−0.2;0)		
MRC									
Mean (SD)	70.6 (13.3)	76.4 (4.7)	−5.8 (11.6)	**<.00**	74.3 (7.3)	77.5 (4.8)	−3.1 (5.6)	**<.000**	.15
Median (IQR)	75 (67.5‐78)	78 (76‐80)	−3 (−5;‐0.5)		77 (72‐80)	80 (77.5‐80)	−1 (−4;0)		
MRC ≤70, n (%)	8 (28.6)	2 (7.1)	–	.**008**	14 (23.7)	4 (7.1)	–	.**0005**	.55

Note

CG were assessed in 87 patients at T0.

Significant *p*‐values are in bold.

Abbreviations: CG, Cryoglobulins; DN4, Douleur Neuropathique 4; INCAT, Inflammatory Neuropathy Cause and Treatment; MRC, Medical Research Council; NPSI, Neuropathic Pain Symptom Inventory; Qol, Quality of life; SSS, Sensory Sum Score.

* CG‐ n = 56 at T1 (3 patients dropped out).

## 4 DISCUSSION

Extrahepatic manifestations are responsible for a portion of the morbidity and mortality related to HCV infections, and have even been regarded as contraindications to treatment in the era of IFN‐based therapies, or a matter of concern for drug‐drug interactions. Moreover, studies exploring the effects of successful antiviral therapies on these comorbidities are lacking. In the present prospective study, we document for the first time an improvement of motor and sensory neurological functions in a cohort of HCV‐positive patients after viral eradication with DAAs. A global assessment of neurological function and impairment was carried out, combining validated sensory and motor neuropathy scales, disability, together with CMAP and SNAP amplitudes, motor and sensory conduction velocities by standard nerve conduction studies. This is to our knowledge the largest systematic study assessing the effects of any anti‐viral therapy on HCV‐associated neuropathy, and the only one reporting on both CG‐ and CG+patients.^5^, ^13^ A recent metanalysis of all 4 eligible trials, the largest of which run in 59 patients randomized to rituximab versus conventional therapy,^33^ included only patients with HCV‐related cryoglobulinemia and none reported on a global neuropathy assessment with pre‐ and post‐treatment measurements. Furthermore, there are at present no reliable studies evaluating the treatment of non‐cryoglobulinemic neuropathies associated with HCV infection.

In our study, at recruitment, gold standard electrophysiological analyses together with clinical examination disclosed a predominant symmetrical sensori‐motor neuropathy, consistent with axonal damage, in approximately one fourth of HCV+patients and neuropathic pain in almost 40% of them, while 16% complained of moderate to severe sensory impairment (SSS), possibly reflecting associated small fibre neuropathy. Furthermore, one fourth of the patients had severe impairment of muscle strength. Prevalence values were higher, though not significantly so, in the presence of cryoglobulinemia, a worsening component in all neurological findings.^29^ CG+patients were slightly older than CG‐ patients, possibly in line with the well‐established development of immune senescence and autoimmunity with aging.^34^

In line with previous reports,^35^ severity of liver fibrosis, viral genotype and viral load were not associated with neurological findings. However, a greater neurological functional impairment was associated with a worse hepatological prognostic index in 4 patients, suggesting an added component related to hepatic involvement, as for kidney disease or cardiovascular complications.^16^, ^17^, ^36^

A new era in the natural history of HCV infection started in 2013 with the introduction of DAA, achieving approximately 95% viral eradication rate with reduced side effects compared to previous treatments.^14^, ^37^ In our study, approximately 1 year after recruitment, DAA‐induced viral eradication significantly improved all neurological scores, in terms of neuropathic pain, impairment, disability and even normalized electrophysiological alterations in 4 patients, independently of clinical and liver disease characteristics, as shown by recent studies assessing the effects of DAAs in other extrahepatic complications.^16^, ^17^, ^38^ Moreover, a trend of increase of both motor and sensory nerve amplitudes was detected in the whole subgroup of patients with altered electroneurography at recruitment, however reaching or approaching statistical significance only for a few electroneurographic parameters. On the contrary, no increase in nerve conduction velocity was detected, possibly reflecting axonal re‐growth preceding myelination. In fact, it is established that regenerated, still non myelinated fibres, have got a low conduction velocity undetectable by standard nerve conduction studies.^39^, ^40^ Notably, significant neurological improvement was detected also in GC+patients, who presented with worse sensory impairment, despite the fact that cryoglobulins are a negative predictive factor for neuropathy, associated with more severe involvement at histometrical analysis,^8^ reflecting additional pathogenetic mechanisms and, possibly, inducing permanent nerve damage.^8^, ^41^

There is a growing body of literature on the association between HCV infection and reduced quality of life, impacting all aspects of function.^3^, ^38^, ^42^ Cognitive impairment, fatigue, depression, independently of risk behaviours, have the greatest weight. In our study, viral eradication with well tolerated DAAs, improved quality of life, possibly in relation to improved neurological function, as assessed by evaluation of mobility, self‐care, usual activities, pain or discomfort, anxiety and depression.

HCV‐related neuropathies include symmetrical axonal sensorimotor neuropathy, accounting for over 50% of cases, distal symmetric painful small‐fibre neuropathy with predominantly sensory features, mononeuritis multiplex, pure motor polyneuropathy or, rarely, demyelinating and autonomic neuropathies.^12^, ^43^, ^44^ Severe peripheral neuropathy, although uncommon, is also described.^8^, ^18^, ^43^ Our study confirms the high prevalence of axonal neuropathy both in CG+and CG‐ HCV‐positive patients, strengthen by the dimension of the cohort examined compared to previous studies.^7^, ^8^

We do not provide evidence on mechanisms underlying neurological improvement. Indeed, pathogenic mechanisms of nerve damage are speculative and likely multifactorial. These include cryoglobulin deposition and vasculitis of epineurial, perineurial vessels and *vasa nervorum*, intravascular cryoglobulin deposition and vessel deposits of HCV‐containing immune complexes causing ischaemia, binding of C1q protein and complement activating pathways.^44^ It has also been suggested that HCV may have a direct pathogenetic role through direct, cytopathic effect^8^, ^44^, ^45^ or by immune‐mediated mechanisms such as immune complex‐induced changes of the epineural vessels, inflammatory lymphocyte infiltrates of epineurium and endoneurium.^44^, ^46^ At central level, HCV replication has been demonstrated in brain microvascular endothelial cells, with release of infectious virus, conformational changes of endothelium, viral and cytokine passage across the blood‐brain barrier, together with microglial activation inducing a state of neuroinflammation.^47^, ^48^ Furthermore, recent studies indicate that viral eradication with DAAs is associated with a significant improvement in endothelial function, vascular distensibility, reduction in insulin resistance and improved glycaemic control.^15^, ^16^, ^17^, ^48^, ^49^ Improved metabolic and vascular functions could thus involve nerve microvasculature, preventing ischaemic damage, curbing endothelial activation and the local inflammatory response.^43^

According to international guidelines, from June 2018 all patients with HCV infection should be considered for treatment with DAAs, prioritizing those with symptomatic cryoglobulinemic vasculitis, extensive liver fibrosis and stage 4‐5 CKD.^36^, ^50^ In line with this recommendation, our report strengthens the indications for early antiviral therapy, independently of the severity of liver disease, on the basis of the risk of developing serious extrahepatic complications, including neuropathy, with a potentially rapidly progressive course.^18,19^

The present study has strengths and limitations. The number of subjects examined prospectively is the largest assessed so far for neurological implications of HCV eradication, in a single centre and regardless of cryoglobulinemia. Limitations include lack of assessment of small sensory fibres, which requires more sophisticated tests as pain related‐evoked potential and skin biopsy, and the lack of a control group, ethically unconceivable.

This work adds to recent reports of significant benefits after successful eradication of HCV by well tolerated DAA regimens, broadening the spectrum of patients eligible for therapy; HCV‐related neuropathy should be considered a major indication for treatment even in the absence of liver disease. Long term follow‐up studies exploring whether sustained virological response is associated with wider, further neurological improvement are welcome.

## CONFLICTS OF INTEREST

None of the authors has any conflict of interest to disclose.

## AUTHOR CONTRIBUTIONS

MMZ was responsible for conception and design of the study, clinical examination, data analysis and wrote the manuscript. CM and AC were responsible for patient selection and hepatological follow up. CM, FZ, CC performed clinical examination, administered questionnaires and collected data. DC, ES, BF performed electrophysiological measurements and analysed data. LC, FC performed statistical analyses. MP and GMS oversaw research and contributed to the discussion. All the authors gave the final approval to the submission of the manuscript. MMZ and MP are the guarantors of this work and, as such, had full access to all the data and take responsibility for the integrity of the data and the accuracy of the data analysis.

## Supporting information

Fig S1

## Data Availability

The data that support the findings of this study are available on request from the corresponding author. The data are not publicly available due to privacy or ethical restrictions.
