# Supplementary material for: Peripheral neuropathy after viral eradication with direct‐acting antivirals in chronic HCV hepatitis: A prospective study
Source: Liver Int. 2021 Jul 20;41(11):2611–21. doi: 10.1111/liv.15002 (PMC8596576; doi:10.1111/liv.15002)

**Figure 1 ESM. Electrophysiological data of patients with neuropathy and with normalized**

**value at T1.** Values of CAMP amplitude, MCV and DL for motor nerves (**a**), and of SNAP

amplitude, SCV and DL for sensory nerves (**b**) for each of the 4 patients with lower limb

neuropathy at T0 and T1. Laboratory cut-off value (control mean ± 2SD), dashed line.


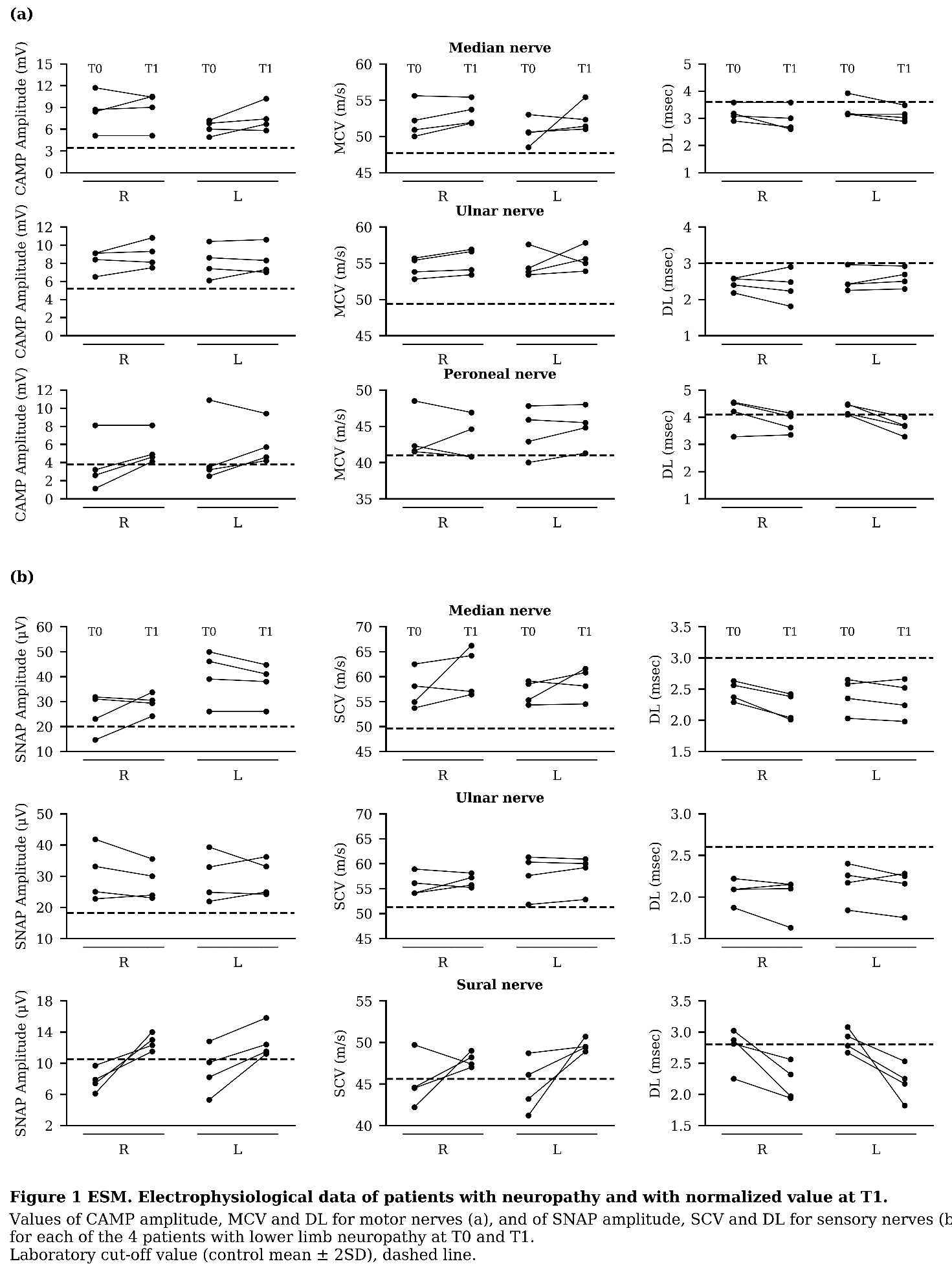

Supplement: Supplementary file 1 — Fig S1 [file LIV-41-2611-s001.docx]
